# Clinical Characteristics and Outcomes of 1894 Women with Peripartum Cardiomyopathy Treated with and Without Levosimendan in Germany

**DOI:** 10.3390/jcdd13030126

**Published:** 2026-03-09

**Authors:** Jan A. Kloka, Alexandra Popescu, Benjamin Friedrichson, Thomas Jasny, Lea V. Blum, Stephanie Noone, Armin Flinspach, Peter Kranke, Kai Zacharowski, Vanessa Neef

**Affiliations:** 1 Department of Anaesthesiology, Intensive Care Medicine and Pain Therapy, University Hospital, Goethe University Frankfurt, Theodor-Stern Kai 7, 60590 Frankfurt, Germany; 2 Intensive Care, Emergency and Pain Medicine, Department of Anaesthesiology, University Hospital Würzburg, 97080 Wuerzburg, Germany

**Keywords:** peripartum cardiomyopathy, obstetrics, healthcare, levosimendan, heart failure, maternal health, cardiogenic shock

## Abstract

Background: Peripartum cardiomyopathy (PPCM) is a rare form of heart failure occurring during the last month of pregnancy or within five months postpartum. While levosimendan is considered beneficial in heart failure and cardiogenic shock, evidence supporting its use in PPCM is limited. This study investigated the prevalence of PPCM in Germany and evaluated outcomes associated with levosimendan use. Methods: Using national data from the German Statistical Office, all pregnant women diagnosed with PPCM and hospitalized between 2009 and 2022 were included. Patients were categorized into groups based on levosimendan treatment. Demographics, treatment modalities, peripartum complications, and transfusion rates were analyzed. Results: The prevalence of PPCM in Germany is 0.01%; 3.60% of patients received levosimendan, mostly after childbirth (61.76%). Peripartum complications and the use of mechanical circulatory support devices were significantly higher in the levosimendan group (*p* < 0.0001). Women in the levosimendan group suffered significantly more often from intrapartum bleeding and increased rates of blood transfusion compared to women in the non-levosimendan group. Conclusions: PPCM is a rare disease in Germany with a prevalence of 0.01%. Despite higher complication rates in women with levosimendan treatment, further studies are necessary to help determine the role and timing of levosimendan in the treatment of women with PPCM.

## 1. Introduction

Especially in western countries, cardiovascular disease is increasingly emerging as a cause of peripartum morbidity and mortality [1]. Peripartum cardiomyopathy (PPCM) is a pregnancy-related form of heart failure with a reduced left ventricular ejection fraction below 45%, for which no other cause of heart failure can be identified. PPCM develops in the peripartum phase, a period leading up to several months after delivery [2]. PPCM may occur globally; however, its prevalence varies among countries, individual national registries and different ethnic groups [3]. In 2020, Sliwa et al. published the results of the first global registry on PPCM as part of the EURObservational Research Program (EORP) hosted by the European Society of Cardiology (ESC) [4]. Data from the ESC reveals a reported prevalence of PPCM in Germany of 1 in 1500 women (0.06%) [5].

The treatment of PPCM follows the principles of standard heart failure therapy, depending on the peripartum phase in which the patient becomes symptomatic due to hemodynamic changes [6]. In cases with severe acute heart failure or cardiogenic shock, mechanical circulatory support such as extracorporeal membrane oxygenation (ECMO) may be necessary [7].

Levosimendan is an inodilator, which enhances myocardial contractility through myofilament calcium sensitization by binding to troponin-C in a calcium concentration-dependent manner and induces peripheral and coronary vasodilation by opening adenosine triphosphate-sensitive potassium channels [8,9]. Although beneficial effects of levosimendan therapy have been described in previous case reports [10,11,12], a recently published small, randomized study demonstrated that levosimendan, however safe, did not provide substantial benefits. The study included 24 women with diagnosed PPCM and levosimendan treatment; however, outcomes did not improve compared to standard treatment [13]. Investigating the value of levosimendan in women with PPCM is lacking; thus, stronger data from larger trials are warranted [14]. With the intention to improve the management of women diagnosed with PPCM, the present study aims to examine clinical characteristics and outcomes in women diagnosed with PPCM over the last 15 years in Germany, using a large database.

## 2. Material and Methods

### 2.1. Inclusion Criteria

In this study, all pregnant women hospitalized in Germany between 1 January 2009 and 31 December 2022 (n = 13,714,325) were screened. In total, 1894 women with PPCM were included in the analyses.

### 2.2. Availability of Data and Materials

German hospitals are required by law to document diagnoses using the International Statistical Classification of Diseases and Related Health Problems (ICD) codes, as well as procedures according to the International Classification of Procedures in Medicine (OPS) codes [15]. The data were stored locally by the German Federal Statistical Office. All analyses were conducted remotely, and individual patient or hospital identifiers were not accessible to the authors. As the data were anonymized by the German Federal Statistical Office, the General Data Protection Regulation was not applicable. Consequently, the requirement for ethical approval and the need for informed consent was waived by the Ethics Committee of the University Hospital Frankfurt (Chair: Prof. Dr. Harder, 2022-766).

### 2.3. Definitions and Data Acquisition

In the dataset, peripartum cardiomyopathy was diagnosed from the last month of pregnancy until five months postpartum (ICD Code O90.3). In addition, cardiomyopathy during any point of pregnancy was also coded as PPCM (O90.3). All age groups and data from 2009 to 2022 were included. More recent data were not available due to accounting aspects and the internal data validation processes of the German Federal Statistical Office. The German Federal Statistical Office processes the data, assesses their validity, and releases them for further scientific analysis.

The data collected included demographics (e.g., age, hospital length of stay (LOS), and pregnancy week), comorbidities (e.g., essential hypertension), complications (e.g., pneumonia), and time points of levosimendan administration (e.g., prepartum or postpartum). Diagnoses were coded according to the 10th revision of the International Classification of Diseases and procedures according to the International Classification of Procedures in Medicine (version 2020). Due to privacy regulations, case numbers of <3 are censored and marked as (*) in tables. The assignment of OPS and ICD-10 codes to the corresponding procedures and diseases can be found in Supplemental Table S1.

### 2.4. Statistical Analysis

Categorical variables are expressed as absolute numbers and percentages. Continuous variables were tested for normality. All considered continuous variables (age, hospital LOS, timing of levosimendan administration and mechanical ventilation) were non-normally distributed. Hence, continuous variables were presented as the median with 25% and 75% quartiles (25; 75% IQR). Group differences between categorical variables were tested for statistical significance with the chi-square test, and for continuous variables with Wilcoxon rank-sum test. Women with PPCM were divided into the levosimendan and non-levosimendan groups. The statistical significance level was set to 5%. Excel 2019 (Microsoft Corp., Seattle, WA, USA) was used for data handling and SAS (Version 9.4M6, SAS Institute Inc., Cary, NC, USA) for statistical analysis.

## 3. Results

**Figure 1:**
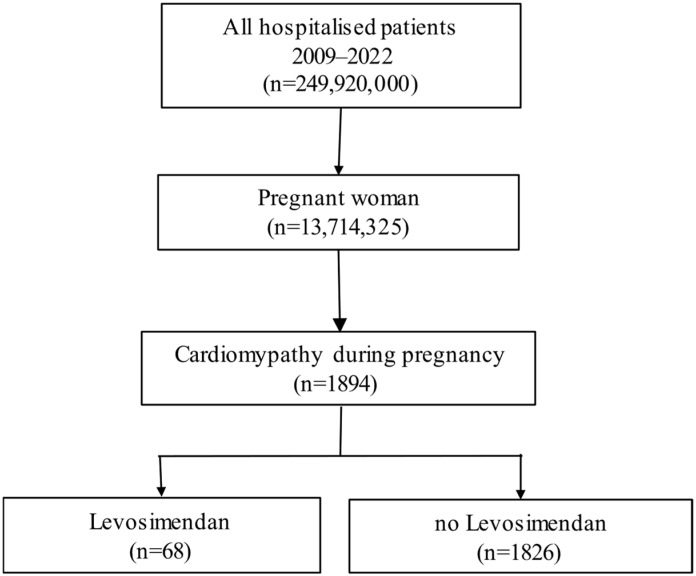
Patient flowchart and patient selection.

In total, 13,714,325 pregnant women were hospitalized between 1 January 2009 and 31 December 2022. In total, 1894 (0.01%) women were with diagnosed PPCM; of these, 68 (3.60%) women received levosimendan (Figure 1).

### 3.1. Demographic Characteristics of Women

The median age of all women with PPCM was 32 (28; 36) years. Existing comorbidities were mostly essential hypertension (11.08%), gestational hypertension (5.86%), gestational diabetes (8.03%) and obesity (6.49%). The mode of delivery was cesarean section in 46.51% of patients, with a median time from hospital admission to delivery of 15 (3; 57) hours (Table 1). For a detailed EUROSCORE-based stratification of cardiac severity see Supplement Table S1.

The baseline characteristics of the women in the levosimendan group and women of the non-levosimendan group are listed in Table 2.

The two groups did not differ in time to presentation, age or pregnancy-related comorbidities. In the levosimendan group, levosimendan was administered mostly after childbirth (61.76%); 38.24% of women received levosimendan before delivery. The median (25; 75% IQR) duration of hospital admission to administration of levosimendan was 27.18 (4.2; 136.28) hours. Most women with levosimendan treatment delivered via cesarean section (36.76%); however, there was no difference between the two study groups. Lastly, hospital LOS was significantly higher in the levosimendan group compared to the non-levosimendan group (437.43 (265.43; 666.82) vs. 209.92 (120; 358.22) h; *p* < 0.0001).

### 3.2. Use of Levosimendan, Complications and Treatment Options

Overall, between 2009 and 2022, the use of levosimendan gradually increased in Germany from 4.41% in 2014 to 14.71% in 2022 (Table 3).

More women in the levosimendan group needed mechanical circulatory support systems (e.g., ECMO) compared to women in the non-levosimendan group (20.59 vs. 1.2%; *p* < 0.0001). The rate of complications, e.g., cardiogenic shock (38.24 vs. 2.57%; *p* < 0.001) and pneumonia (44.12 vs. 15.01%; *p* < 0.0001), as well as admission to the intensive care unit (50 vs. 21.47%; *p* < 0.0001) were significantly higher in the levosimendan group compared to the non-levosimendan group. Significant differences were also found in the occurrence of maternal death between the levosimendan and non-levosimendan groups (4.41 vs. 0.55%; *p* = 0.0002) (Table 4). 

### 3.3. Administration of Blood Products

Women with levosimendan treatment experienced significantly more often intrapartum bleeding compared to women without levosimendan treatment (7.35 vs. 1.64%; *p* = 0.0006). In addition, blood products were administered more often in the levosimendan group compared to the non-levosimendan group. Specifically, allogeneic red blood cells (RBCs) were transfused significantly more often in women with levosimendan treatment compared to women without levosimendan (64.71 vs. 14.95%; *p* < 0.0001). In addition, massive blood transfusion (defined as >5 RBCs) was needed more frequently in women with levosimendan compared to women without levosimendan (23.53 vs. 2.41%; *p* < 0.0001) (Table 5).

Further analyses demonstrate that levosimendan is mostly administered in women with PPCM in hospitals with an annual delivery rate of 1501–3000 births (Supplemental Table S2).

## 4. Discussion

This retrospective study includes a cohort of 13,714,325 women hospitalized between 1 January 2009 and 31 December 2022 in Germany. The numbers indicate that PPCM is a rare disease in Germany, with a prevalence of 0.01%. Of these, 3.60% of patients received levosimendan, mostly after childbirth (61.76%). The rate of peripartum complications and use of mechanical circulatory support systems were significantly higher in the levosimendan group compared to the non-levosimendan group. Last, women with levosimendan suffered significantly more often from intrapartum hemorrhage and had increased rates of blood transfusions compared to women without levosimendan treatment.

PPCM is a devastating form of cardiac failure during pregnancy or in the early postpartum period, despite the absence of known preexisting cardiac dysfunction [16]. Regarding the timing of development, there are also cases where the disease occurs earlier in pregnancy or more than 5 months after delivery [4,17,18].

The prevalence of PPCM in the present study was 0.01% over the past 15 years in Germany, which is lower compared to recently published studies. The estimated global incidence of PPCM is around 1 every 2000 deliveries, with wide regional differences [19]. In African countries, PPCM prevalence surpasses one case per 1000 births. Caucasian women seem to be less affected; so far, there are reported incidences of 1 in 1500 (0.06%) in Germany and 1 in 5719 (0.02%) in Sweden [5].

Regarding the treatment of PPCM, a few randomized trials have evaluated therapies for PPCM; however, none of these studies had conclusive results. Thus, current management is largely extrapolated from guideline-directed medical treatment for non-ischemic dilated cardiomyopathy and other forms of heart failure with a reduced ejection fraction [2,6]. Levosimendan has been reported to improve hemodynamics, symptoms, and clinical outcomes in patients with decompensated heart failure [20]. To date, there are only a few studies and limited data on the efficiency and value of levosimendan in patients with PPCM. So far, in women with PPCM, beneficial effects of levosimendan therapy have been described in multiple case reports [10,11,12,21,22]. A small, randomized study in 2004 included 24 women with PPCM and revealed the safety of levosimendan, but did not show substantial benefits and did not improve outcomes compared to women with conventional treatment [13]. Another comparison of milrinone and levosimendan in 15 women with PPCM showed comparable hemodynamic improvement with both drugs [23]. Analyses from the present study reveal that overall complications occurred significantly more often in the levosimendan group compared to the non-levosimendan group, e.g., pneumonia (44.12 vs. 15.01%; *p* < 0.0001), pulmonary artery embolism (7.35 vs. 1.2%; *p* < 0.0001), hospital LOS and hours of mechanical ventilation. Recently published data demonstrate that 60% of all cases of cardiogenic shock during or shortly after pregnancy are caused by PPCM [24]. Women with levosimendan were more severely ill, as the drug is primarily administered in the setting of markedly reduced cardiac function. Therefore, the higher rates of complications observed in this group are most likely attributable to the underlying severity of cardiac dysfunction rather than to a direct effect of levosimendan therapy. In addition, significant increased maternal death in women with levosimendan may be related to the severity of left ventricular dysfunction and the extent of hemodynamic compromise at presentation. Patients requiring levosimendan typically represent a subgroup with more advanced heart failure or cardiogenic shock, which is independently associated with a worse prognosis.

Temporary mechanical circulatory support with an intra-aortic balloon pump, percutaneous ventricular assist device therapy, and ECMO treatment have been used successfully in PPCM and should be considered early in patients with hemodynamic instability [25,26]. In the present study, cardiogenic shock occurred significantly more often in the levosimendan group compared to the non-levosimendan group, which may be explained by the disease severity of women in the levosimendan group (Elixhauser Comorbidity Index (25; 75 IQR) (12 (8; 18.5) vs. 6 (0; 12)); *p* < 0.001). Mechanical circulatory support, e.g., with ECMO treatment, was used more often in the levosimendan group compared to the non-levosimendan group (*p* < 0.0001). The higher proportion of women requiring mechanical circulatory support in the levosimendan group is attributable to the greater baseline disease severity in these women. The increased need for mechanical circulatory support in this group likely reflects the underlying severity of illness rather than a direct adverse effect of levosimendan itself. The severity of the disease is likely also the reason for the other observed outcome, e.g., in terms of blood product use. It is noteworthy to mention that the analyzed groups had different sample sizes, due to the overall low prevalence of PPCM. Therefore, the results should be interpreted with caution, as the imbalance may increase the risk of misleading conclusions.

The timing of levosimendan administration data from the present study reveal that levosimendan was given 27.18 (4.2; 136.28) hours after hospital admission, a median of 17 h after childbirth. In cardiac surgical patients with reduced ventricular function undergoing coronary artery bypass grafting, early (preoperatively) treated patients had significantly lower in-hospital mortality (preop vs. intraop. vs. postop = 16.7% vs. 33.3% vs. 42.3%) as well as overall complication rate compared to intra- or postoperatively treated patients [27]. In cases where inotropic support is required during pregnancy, levosimendan is generally considered safe during pregnancy [28]. Despite the fact of an increased disease severity in women with levosimendan treatment in the present study, it might be assumed that earlier levosimendan administration could improve outcomes in women with PPCM or cardiogenic shock. As data on the timing of levosimendan in women with PPCM during pregnancy are scarce and considering the substantial mortality rate, more robust studies on this aspect are urgently needed.

According to 2025 ESC guidelines on the “Treatment of heart failure during pregnancy”, PPCM treatment is divided into women with severe heart failure/cardiogenic shock and women with non-severe heart failure/cardiogenic shock [29].

Pregnant women presenting with acute heart failure require urgent hospital admission. These patients should be referred to an expert center with established advanced heart failure care, including mechanical circulatory support. In case of cardiogenic shock, recommended inotropic agents include levosimendan [30], dobutamine and milrinone. Levosimendan is administered as a continuous infusion without an initial loading dose [29]. As mentioned above, the use of levosimendan is considered safe during pregnancy. However, the limitations are the potential for hypotension/arrhythmias (requiring careful monitoring) and there are concerns about breastfeeding (metabolites in milk). Thus, this drug necessitates caution and expert management [28].

In non-severe PPCM in the postpartum period, recommended medical therapy consists of heart failure disease-modifying drugs. These include angiotensin-converting enzyme inhibitors (ACEIs), angiotensin receptor/neprilysin receptor inhibitors (ARNIs), mineralocorticoid receptor antagonist (MRA), sodium-glucose cotransporter-2 (SGLT2) inhibitors, beta-blockers and diuretics. In addition, ivabradine can be added in selected PPCM patients. During pregnancy, ACEIs and ARNIs are not recommended due to fetotoxicity. Instead, a combination of hydralazine and nitrates can be used. Also, SGLT2 inhibitors and ivabradine are not recommended during pregnancy and in breastfeeding mothers, due to the lack of safety data. Advanced heart failure therapies in PPCM include cardiac resynchronization therapy, cardioverter defibrillators, left ventricular assist devices or cardiac transplants [29].

The pathophysiology of PPCM is related to the 16 kDa prolactin. The increase in prolactin leads to endothelial damage and impairs cardiomyocyte function. Based on this mechanism, bromocriptine, a dopamine agonist that inhibits prolactin production, has been proposed as a potential treatment for PPCM. The drug is recommended in PPCM patients with severe left ventricular systolic dysfunction or those unwilling to breastfeed [31]. Based on evidence from recent studies, the ESC guidelines recommend adding bromocriptine to treat acute PPCM, especially in severe cases. Thus, bromocriptine treatment may be considered in addition to optimal heart failure treatment to enhance the recovery of left ventricular function in women with PPCM (class II b recommendation) [29]. Patients taking bromocriptine are advised to take preventative anticoagulants due to its pro-thrombotic nature [29]. However, in a recent meta-analysis including 11 studies and more than 1500 participants, no increase in thromboembolic events using bromocriptine was observed [31].

Women in the levosimendan group suffered significantly more often from intrapartum hemorrhage, RBC transfusion rate and massive blood transfusion compared to the non-levosimendan group (*p* < 0.0001). Maternal hemorrhage is a major driver for increased RBC transfusion rates [32]. It is noteworthy to mention that, on one hand, postpartum hemorrhage followed by massive blood transfusion may mask the diagnosis of PPCM or worsen the decompensated heart failure. On the other hand, mechanical circulatory support devices require anticoagulation, which may aggravate intrapartum hemorrhage when used prior to delivery [33]. In situations of massive intrapartum hemorrhage, the use of cell salvage is recommended [34]. In the present study, cell salvage was used in 4.41% of all bleeding women with PPCM and levosimendan treatment. This is more than previously published in >300,000 women with peripartum hemorrhage [35]—however, still too little.

Last, the data used are collected for reimbursement purposes; thus, they are subject to validation by the Medical Service of the Health Insurance Companies, which ensures a defined level of data quality and plausibility. Diagnoses recorded in billing datasets are generally based on prior diagnostic procedures that must be performed to justify remuneration, implying that relevant clinical assessments have taken place before a diagnosis is coded. Importantly, reimbursement data should not necessarily be viewed as a substitute for prospectively collected clinical data (e.g., PPCM registry), but rather as a complementary data source. Their additive use alongside prospectively collected datasets can enhance external validity, increase statistical power, and support the triangulation of findings.

### Limitations

To the best of our knowledge and based on an extensive literature review, this is the largest study of women with PPCM treated with levosimendan over a 15-year period in Europe. However, there are some limitations of the present study. One of the main limitations of the present study is its retrospective nature and misclassification bias due to the simple use of ICD codes and secondary reimbursement data. Reimbursement data have a correlation with the medical cases in the hospital [36]. However, it cannot be entirely precluded that certain conditions or events might be either over- or under-represented due to reimbursement considerations. Nonetheless, there exists an increased incentive for consistent and accurate documentation, as the medical service of the health insurance funds conduct audits on hospital reimbursements. Fraudulent documentation could negatively impact compensation or even lead to penalties. The parameters chosen for this study were based on their high medical relevance, aiming to minimize the occurrence of coding errors. Due to the large sample size, possibly misreported data should be largely counterbalanced. Data were collected in a structured and representative manner according to the Declaration of Helsinki and according to the STROBE Guideline. Laboratory findings or medications used (e.g., bromocriptine) are not coded for reimbursement and are therefore not available for analysis. In addition, selection bias—patients included are those who interact with the healthcare system and have the resource to do so—and detection bias—more doctors’ visits, better compliance, etc., result in more diagnoses—have to be mentioned. Also, echocardiographic data on left ventricular function and dimensions and medication intake are not available. The latter limits the causal attribution of the intervention in view of the observed outcome.

The unequal distribution of sample sizes between the two groups can substantially limit the statistical validity of the analysis and lead to erroneous conclusions, e.g., that levosimendan should be avoided in acute heart failure. However, disease severity may explain adverse events in the levosimendan group. In particular, statistical power is reduced, increasing the likelihood that true effects remain undetected. Furthermore, estimates of means and variances in the smaller group tend to be less stable and more susceptible to the influence of outliers. Many statistical tests also assume homogeneity of variances, and violations of this assumption in the presence of unequal group sizes may lead to biased *p*-values. Last, phenotypic heterogeneity has to be addressed, as cardiomyopathy during any point of pregnancy was coded as PPCM. This definition bundles any etiology of cardiomyopathy as peripartum cardiomyopathy.

## 5. Conclusions

The prevalence of PPCM in Germany is low (0.01%). Despite emerging interventional and pharmacological treatment options, PPCM is a life-threatening condition. In this study, the administration of levosimendan compared to conventional therapy did not improve outcomes in patients with PPCM. In addition, the rate of intrapartum hemorrhage and the administration of blood products were higher in the levosimendan group compared to the non-levosimendan group. Future prospective studies or patients’ registries providing additional relevant data to correlate treatment outcome with interventions are urgently needed in order to increase patient safety in the management of women with PPCM.

## Figures and Tables

**Table 1:** Characteristics of women diagnosed with PPCM from 2009 to 2022.

	PPCM	
Total patients, n	1894	
Age, median years (Q1; Q3)	32 (28; 36)	
**Disease severity**		
Elixhauser Comorbidity Index, median (Q1; Q3)	6 (0; 11)	
Length of stay; median hours (Q1; Q3)	217 (121; 366)	
Ventilation; median hours (Q1; Q3)	27 (8; 96)	
**Timing**		
Hospital admission to delivery; median hours (Q1; Q3)	15 (3; 57)	
**Age groups**		
15–19; n, %	44	2.32
20–24; n, %	180	9.50
25–29; n, %	386	20.38
30–34; n, %	583	30.78
35–39; n, %	497	26.24
40–44; n, %	180	9.50
45–49; n, %	18	0.95
50–54; n, %	6	0.32
**Pregnancy week**		
<5; n, %	0	0.00
15–13; n, %	4	0.21
14–19; n, %	5	0.26
20–25; n, %	*	*
26–33; n, %	193	10.19
34–36; n, %	223	11.77
37–41; n, %	605	31.94
>41; n, %	41	2.16
**Birth mode**		
Vaginal delivery; n, %	264	13.41
Cesarean section; n, %	881	46.51
**Comorbidities**		
Essential hypertension; n, %	210	11.08
Gestational hypertension; n, %	111	5.86
Diabetes during pregnancy (pre-existing); n, %	29	1.53
Gestational diabetes; n, %	152	8.03
Nicotine abuse; n, %	22	1.16
Obesity; n, %	123	6.49
Grade I; n, %	15	0.79
Grade II; n, %	10	0.52
Grade III; n, %	*	*

* Institutionally anonymized <3 cases.

**Table 2:** Characteristics of women with PPCM treated with or without levosimendan.

	Non-Levosimendan		Levosimendan		*p*-Value
Total patients, n	1826		68		
Age, median years (Q1; Q3)	32 (28; 36)		32.5 (27.5; 37)		0.7511
**Disease severity**					
Elixhauser Comorbidity Index, median (Q1; Q3)	6 (0; 12)		12 (8; 18.5)		<0.0001
Length of Stay; median hours (Q1; Q3)	209.92 (120; 358.22)		437.43 (265.43; 666.82)		<0.0001
Ventilation; median hours (Q1; Q3)	25 (8; 75)		120 (16.5; 330.5)		0.0001
**Timing of levosimendan administration**					
Postpartum	n.a.		42	61.76	
Prepartum	n.a.		26	38.24	
Hospital admission to levosimendan; median hours (Q1; Q3)	n.a.		27.18 (4.2; 136.28)		
Hospital admission to delivery; median hours (Q1; Q3)	15.8 (3.03; 56.55)		10.84 (3.5; 71.35)		0.8064
Levosimendan to delivery; median hours (Q1; Q3)	n.a.		−32.51 (−131.5; −13.63)		
**Age groups**					
15–19; n, %	*	*	*	*	
20–24; n, %	173	9.47	7	10.29	
25–29; n, %	369	20.21	17	25.00	
30–34; n, %	564	30.89	19	27.94	
35–39; n, %	480	26.29	17	25.00	
40–44; n, %	175	9.58	5	7.35	
45–49; n, %	*	*	*	*	
50–54; n, %	*	*	*	*	
**Pregnancy week**					
<5; n, %	0	0.00	0	0.00	
15–13; n, %	4	0.22	0	0.00	
14–19; n, %	5	0.27	0	0.00	
20–25; n, %	*	*	*	*	
26–33; n, %	187	10.24	6	8.82	
34–36; n, %	215	11.77	8	11.75	
37–41; n, %	593	32.48	12	17.65	
>41; n, %	41	2.25	0	0.00	
**Birth mode**					
Vaginal delivery; n, %	254	13.91	10	14.71	0.8524
Cesarean section; n, %	856	46.88	25	36.76	0.1006
**Comorbidities**					
Essential hypertension; n, %	201	11.01	9	13.24	0.5657
Gestational hypertension; n, %	107	5.86	4	5.88	0.9938
Diabetes during pregnancy (pre-existing); n, %	29	1.59	0	0.00	0.4241
Gestational diabetes; n, %	148	8.11	4	5.88	0.5077
Nicotine abuse; n, %	22	1.20	0	0.00	0.3626
Obesity; n, %	120	6.65	3	4.41	0.4779
Grade I; n, %	15	0.82	0	0.00	0.4530
Grade II; n, %	10	0.55	0	0.00	0.5406
Grade III; n, %	*	*	*	*	0.2576

* Institutionally anonymized <3 cases. Obesity grade 1: body mass index (BMI): 30.0–34.9 kg/m^2^, grade II: BMI 35.0–39.9 kg/m^2^, grade III: BMI ≥ 40.0 kg/m^2^.

**Table 3:** Use of levosimendan in Germany in women with PPCM from 2009 to 2022.

	Total		Non-Levosimendan		Levosimendan	
	n	%	n	%	n	%
**Year**						
2009	92	4.86	*	*	*	*
2010	91	4.8	*	*	*	*
2011	102	5.39	*	*	*	*
2012	106	5.6	*	*	*	*
2013	116	6.12	*	*	*	*
2014	143	7.55	140	7.67	3	4.41
2015	148	7.81	*	*	*	*
2016	172	9.08	166	9.09	6	8.82
2017	144	7.6	131	7.17	13	19.12
2018	172	9.08	165	9.04	7	10.29
2019	158	8.34	153	8.38	5	7.35
2020	178	9.4	169	9.26	9	13.24
2021	152	8.03	146	8.00	6	8.82
2022	120	6.34	110	6.02	10	14.71

* Institutionally anonymized <3 cases; obesity grade 1: body mass index (BMI): 30.0–34.9 kg/m^2^, grade II: BMI 35.0–39.9 kg/m^2^, grade III: BMI ≥ 40.0 kg/m^2^.

**Table 4:** Complications and mechanical circulatory support in women with PPCM treated with or without levosimendan.

	Non-Levosimendan		Levosimendan		*p*-Value
**Mechanical Circulatory Support**					
ECMO treatment; n, %	22	1.2	14	20.59	<0.0001
VA; n, %	15	0.82	12	17.65	<0.0001
VV; n, %	7	0.38	*	*	0.0026
Impella; n, %	*	*	*	*	<0.0001
IABP; n, %	*	*	*	*	0.2747
Coronary intervention; n, %	7	0.38%	0	0.00%	0.6090
Stenting; n, %	4	0.22%	0	0.00%	0.6992
**Complications**					
ICU admission; n, %	392	21.47	34	50	<0.0001
Death; n, %	10	0.55	3	4.41	0.0002
Fluid and electrolyte disorders; n, %	399	21.85	33	48.53	<0.0001
Delirium; n, %	17	0.93%	7	10.29%	<0.0001
Cardiogenic shock; n, %	47	2.57%	26	38.24%	<0.0001
Pneumonia; n, %	274	15.01	30	44.12	<0.0001
Postpartum acute renal failure; n, %	95	5.20	9	13.24	0.0043
Stroke; n, %	16	0.88	5	7.35	<0.0001
Pulmonary artery embolism, n, %	22	1.20	5	7.35	<0.0001
ARDS; n, %	19	1.04	6	8.82	<0.0001
Cardiopulmonary resuscitation; n, %	40	2.19	9	13.24	<0.0001
Hyperdynamic cardiac arrest; n, %	9	0.49%	6	8.82%	<0.0001
Asystole; n, %	33	1.81%	12	17.65%	<0.0001
Pulseless electrical activity; n, %	0	0.00%	0	0.00%	
Arrythmia; n, %	56	3.07%	6	8.82%	0.0088
Miscarriage (child born dead); n, %	17	0.93	0	0	0.4241

* Institutionally anonymized <3 cases.

**Table 5:** Number of administered blood products in women with and without levosimendan.

	Non-Levosimendan		Levosimendan		*p*-Value
**Blood products**					
Red blood cells	273	14.95	44	64.71	<0.0001
Fresh Frozen Plasma	50	2.74	13	19.12	<0.0001
Platelets	75	4.11	16	23.53	<0.0001
Prothrombin complex concentrate	48	2.63	14	20.59	<0.0001
Fibrinogen	91	4.98	14	20.59	<0.0001
Massive blood transfusion	44	2.41	16	23.53	<0.0001
Cell Salvage	11	0.6	3	4.41	0.0003
**Bleeding**					
Prepartum hemorrhage; n, %	9	0.49	0	0.00	0.5617
Intrapartum hemorrhage; n, %	30	1.64	5	7.35	0.0006
Postpartum hemorrhage; n, %	176	9.64	11	16.18	0.0760

## Data Availability

The data on which the results of this study are based are available from the Federal Statistical Office with the restrictions applied. The dataset was used under license for the current study and is therefore not generally accessible. However, the data are available from the corresponding author on reasonable request and with permission from the Federal Statistical Office.

## Supplementary Materials

The following supporting information can be downloaded at: https://www.mdpi.com/article/10.3390/jcdd13030126/s1; Supplement Table S1: ICD-10 and OPS allocations; Supplement Table S2: Treatment of women with PPCM grouped by births per hospital.
