# Supplementary material for: Clinical Characteristics and Outcomes of 1894 Women with Peripartum Cardiomyopathy Treated with and Without Levosimendan in Germany
Source: J Cardiovasc Dev Dis. 2026 Mar 9;13(3):126. doi: 10.3390/jcdd13030126 (PMC13026767; doi:10.3390/jcdd13030126)
Supplement: Supplementary file 1 [file jcdd-13-00126-s001.zip › jcdd-4126808-supplementary.pdf]

Supplementary Table S1: ICD-10 and OPS allocations

Diagnosis	OPS codes	ICD-10
Vaginal delivery	9-260, 9-261, 9-268	
Caesarean section	5-74, 5-741, 5-749	
Essential hypertension		I10.-
Gestational hypertension		O13
Diabetes during pregnancy		O24.-
Nicotine abuse		F17.2
Obesity		E66.-
Grade I [BMI] 30- <35		E66.00
Grade II [BMI] 35- <40		E66.01
Grade III [BMI] >/= 40		E66.02
Prepartum haemorrhage		O46.-
Intrapartum haemorrhage		O67.-
Postpartum haemorrhage		O72.-
Pneumonia		J12.-, J13, J14, J15.-, J16.-, J17.-*, J18.-, U69.00
Renal failure		N17.-, N19
Postpartum renal failure		O90.4, O90.9
Cardiopulmonary resuscitation	8-771 , 8-772 , 8-779	
Cardiac complications during pregnancy		O75.4, O75.8, O75.9
Death		O95
Miscarriage (child born dead)		Z37.1, Z37.3, Z37.4, Z37.7
Red blood cells	8-800.c	
Pletelets	8-800.6, 8-800.d, 8-800.f,8-800.g, 8-800.h, 8-800.j, 8-800.k,8-800.m, 8-800.n	
Fresh Frozen Plasma	8.812.6-8.812.8	
Prothrombin complex concentrate	8-812.5	
Fibrinogen	8-810.j	
Massive blood transfusion	8-800.1	
Levosimedan	6-004.d	

Supplementary Table S2: Treatment of women with PPCM grouped by births per hospital

	Cardiomyopathy	Levosimedan		Elixhauser Comorbidity Index	VA ECMO		VV ECMO		Hospitals	
	n	n	%	median [Q1;Q3]	n	%	n	%	n	%
**Number of deliveries**										
<500 - 1,500	1717	58	3.38	6 [0-11]	23	1.34	7	0.4	495	53.51
1,501-3,000	161	10	6.21	7 [5-12]	4	2.48	*	*	27	28.72
3,001- >4,500	*	*	*	7,5 [2,5-11]	*	*	*	*	*	*

* Institutionally anonymized <3 cases
